# PBPK-based dose finding for sildenafil in pregnant women for antenatal treatment of congenital diaphragmatic hernia

**DOI:** 10.3389/fphar.2023.1068153

**Published:** 2023-03-14

**Authors:** Julia Macente, Nina Nauwelaerts, Francesca M. Russo, Jan Deprest, Karel Allegaert, Bart Lammens, Rodolfo Hernandes Bonan, Jessica M. Turner, Sailesh Kumar, Andrea Diniz, Frederico S. Martins, Pieter Annaert

**Affiliations:** ^1^ Department of Pharmaceutical and Pharmacological Sciences, KU Leuven, Leuven, Belgium; ^2^ Gynecology and Obstetrics, UZ Leuven, Leuven, Belgium; ^3^ Department of Development and Regeneration, KU Leuven, Leuven, Belgium; ^4^ Department of Clinical Pharmacy, Erasmus MC, Rotterdam, Netherlands; ^5^ BioNotus GCV, Niel, Belgium; ^6^ Mater Research Institute, University of Queensland, Brisbane, QLD, Australia; ^7^ Pharmacokinetics and Biopharmaceutical Laboratory (PKBio), Department of Pharmacy, State University of Maringa, Maringa, Brazil

**Keywords:** physiologically-based pharmacokinetic modelling, PBPK, model informed drug development, MIDD, sildenafil, revatio, congenital diaphragmatic hernia, pregnant women

## Abstract

Sildenafil is a potent vasodilator and phosphodiesterase type five inhibitor, commercially known as Revatio^®^ and approved for the treatment of pulmonary arterial hypertension. Maternal administration of sildenafil during pregnancy is being evaluated for antenatal treatment of several conditions, including the prevention of pulmonary hypertension in fetuses with congenital diaphragmatic hernia. However, determination of a safe and effective maternal dose to achieve adequate fetal exposure to sildenafil remains challenging, as pregnancy almost always is an exclusion criterion in clinical studies. Physiologically-based pharmacokinetic (PBPK) modelling offers an attractive approach for dose finding in this specific population. The aim of this study is to exploit physiologically-based pharmacokinetic modelling to predict the required maternal dose to achieve therapeutic fetal exposure for the treatment congenital diaphragmatic hernia. A full-PBPK model was developed for sildenafil and N-desmethyl-sildenafil using the Simcyp simulator V21 platform, and verified in adult reference individuals, as well as in pregnant women, taking into account maternal and fetal physiology, along with factors known to determine hepatic disposition of sildenafil. Clinical pharmacokinetic data in mother and fetus were previously obtained in the RIDSTRESS study and were used for model verification purposes. Subsequent simulations were performed relying either on measured values for fetal fraction unbound (*fu* = 0.108) or on values predicted by the simulator (*fu* = 0.044). Adequate doses were predicted according to the efficacy target of 15 ng/mL (or 38 ng/mL) and safety target of 166 ng/mL (or 409 ng/mL), assuming measured (or predicted) *fu* values, respectively. Considering simulated median profiles for average steady state sildenafil concentrations, dosing regimens of 130 mg/day or 150 mg/day (administered as t.i.d.), were within the therapeutic window, assuming either measured or predicted *fu* values, respectively. For safety reasons, dosing should be initiated at 130 mg/day, under therapeutic drug monitoring. Additional experimental measurements should be performed to confirm accurate fetal (and maternal) values for *fu*. Additional characterization of pharmacodynamics in this specific population is required and may lead to further optimization of the dosing regimen.

## 1 Introduction

Sildenafil (SIL) is a phosphodiesterase type 5 (PDE5) inhibitor, and used as a potent vasodilator approved by regulatory agencies for the treatment of erectile dysfunction (Viagra^®^) and pulmonary hypertension (PAH) (Revatio^®^) (Ghofrani et al., 2006; FDA, 2018; EMA, 2022). According to the biopharmaceutics classification system (BCS) SIL is a class 2 drug, rapidly absorbed, reaching T_max_ in approximately 1 h and is highly bound to plasma protein (∼96%). It is mainly metabolized by cytochrome P450 (CYP) enzymes, primarily by CYP3A4 into the active metabolite N-desmethyl-sildenafil (DMS). SIL half-life is between 3 and 4 h for doses of 25–200 mg (Nichols et al., 2002; Zane et al., 2015).

There is evidence that, when administered in pregnancy, SIL inhibits the vasoconstriction in spiral arteries in uterus and increases the fetal and uteroplacental blood flow (Dastjerdi et al., 2012). For these reasons, SIL has been clinically investigated for the treatment of several pregnancy complications, including intrauterine growth restriction, preeclampsia, and fetal distress during labor (de Bie et al., 2022). The maternal administration of SIL during pregnancy is also being evaluated for antenatal treatment of congenital diaphragmatic hernia (CDH) (Luong et al., 2011; Russo et al., 2019a). CDH occurs when the diaphragm is not fully closed during fetal development and leads to pulmonary hypoplasia, pulmonary hypertension and cardiac dysfunction (Deprest et al., 2014). In animal studies, transplacental treatment with SIL has been shown to prevent the lung vascular changes that lead to pulmonary hypertension after birth (Russo et al., 2016; Kashyap et al., 2019). However, the optimal maternal dose required to achieve safe and effective fetal exposure remains elusive. It is well known that pregnancy leads to pronounced changes in physiology as well as pharmacokinetics (PK) compared with non-pregnant women (Abduljalil et al., 2012; Szeto et al., 2021). Unfortunately, clinical (PK) data in this vulnerable population are virtually non-existent, also because pregnancy is a very common exclusion criterion during clinical trials.

In recent years, non-clinical computational tools have already shown their utility for simulating PK in special populations, thereby for instance taking in account the ontogeny of proteins relevant to drug disposition. Such *in silico* tools ultimately allow the assessment of drug exposure for a given dosing regimen. More recently, Model Informed Drug Development (MIDD) has emerged as a powerful alliance for regulatory decisions on drug development. MIDD avoids unnecessary exposure in clinical studies with special populations by predicting drug exposure and enables understanding the PK and pharmacodynamics (PD) for example, in pregnant women (Dallmann et al., 2018; Wang et al., 2019; Kuemmel et al., 2020; Coppola et al., 2021).

MIDD encompasses a spectrum of approaches, one of which is Physiologically-based Pharmacokinetic (PBPK) modeling (Darwich et al., 2006). PBPK modelling is the bottom-up computational approach where the association of the physiologic description of the organism with the physicochemical characteristics of the drug (or chemical) results in plasma or tissue concentration time profiles (Kuepfer et al., 2016). PBPK models allow to simulate different (patho)physiologic conditions, while for instance considering ontogeny of protein expression/function with progressing age of the virtual patients. PBPK modelling can thus help understand the PK drug behavior in the human body without the need to conduct (extensive) clinical studies in special populations (Kuepfer et al., 2016). Therefore, the aim of this work is to develop a PBPK model to predict the required maternal dose to achieve therapeutic fetal exposure for the treatment of congenital diaphragmatic hernia.

## 2 Materials and methods

### 2.1 PBPK model development

#### 2.1.1 PBPK in the non-pregnant population

The Simcyp^®^ simulator version 21 (Simcyp Ltd, Sheffield, UK) was used to develop the PBPK model in reference volunteer individuals and subsequently in pregnant women.

SIL and DMS physicochemical and *in vitro* PK properties were obtained from literature and are summarized in Table 1. The oral absorption was described using the Advanced Dissolution, Absorption and Metabolism (ADAM) model, the effective permeability (P_*eff*_) was estimated using the Mechanistic Permeability (MechPeff) model. The volume of distribution at steady state (V_ss_) was calculated using the Rodgers and Rowland method. The Kp Scalar was adjusted to match with the volume of distribution reported in literature (Nichols et al., 2002). Based on experiments with human liver microsomes (HLM) (Zane et al., 2015), SIL is metabolized into the main active metabolic DMS majorly by CYP3A4, and to a lesser extent by CYP3A5 > CYP3A7. Further proportional scaling was applied to intrinsic clearance reported by Zane et al. (2015) with a factor of 1.46 to the initial values for their sum to match total intrinsic clearance (314 μL/min/mg protein) as retro-gradually calculated based on *in vivo* clearance values (Milligan et al., 2002; Muirhead et al., 2002). For the active metabolite DMS the first order absorption model was used. The fraction absorbed (*fa*) and the first-order absorption rate constant (k_a_) were predicted based on the polar surface area (PSA) and number of hydrogen bond donors (HBD). In addition, the volume of distribution at steady state (V_ss_) was calculated using the Rodgers and Rowland method. The *in vivo* hepatic clearance of DMS was set at 40 L/h as derived from *in vivo* PK data (Muirhead et al., 2002; Nichols et al., 2002). Renal clearance of DMS was assumed to be negligible (Walker et al., 1999; Hyland et al., 2001).

**TABLE 1:** Summary of the input data for the PBPK model development of sildenafil and N-desmethyl-sildenafil.

**Sildenafil**		
** Parameter (unit)**	**Value**	**Reference**
MW (g/Mol)	474.58	DrugBank
LogP	2.7	Ronique Gobry et al. (2000)
Compound type	Monoprotic base	
pKa	6.78	Ronique Gobry et al. (2000)
**Absorption**		
Model	ADAM	
P_*eff*_ (10^−4^ cm/s)	4.48	Predicted using P_app, MechPeff_
Solubility (mg/mL)	3.5	DrugBank
**Distribution**		
Model	Full PBPK	
V_ss_ (L/Kg)	1.15	Predicted with Rodgers and Rowland method
*f*_*u*_	0.036	Walker et al. (1999)
B:P	0.6	European Medicines Agency (2005)
K_p Scalar_	1.79	
**Elimination**		
CL_int_ CYP3A4 (μL/min/mg)	274.4	Zane et al. (2015)
CL_int_ CYP3A5 (μL/min/mg)	32.6	Zane et al. (2015)
CL_int_ CYP3A7 (μL/min/mg)	7.19	Zane et al. (2015)
f_umic_	0.97	Zane et al. (2015)
**N-Desmethyl-Sildenafil**		
MW (g/Mol)	460.6	PubChem
LogP	1.78	DrugBank
Compound type	Monoprotic base	
pKa	6.78	Ronique Gobry et al. (2000)
**Absorption**		
Model	First order	
f_a_	0.38	Predicted using PSA and HBD
k_a_ (h^−1^)	0.14	Predicted using PSA and HBD
PSA	126	PubChem
HBD	2	PubChem
**Distribution**		
Model	Full PBPK	
V_ss_ (L/Kg)	0.26	Predicted with Rodgers and Rowland method
f_u_	0.036	Walker et al. (1999)
B:P	0.6	European Medicines Agency (2005)
K_p Scalar_	1.78	Predicted
**Elimination**		
CL_iv_ (L/h)	40	Predicted
**Pregnancy PBPK model**		
CL_PDM_ (L/h/mL)	0.0038168	Calculated from CL_cot_ Hoeben et al. (2018)
CL_PDF_ (L/h/mL)	0.0038168	Calculated from CL_cot_ Hoeben et al. (2018)
*fu*_plasma_ predicted in fetus	0.044	Predicted with Simcyp Simulator
*fu*_plasma_ in fetus	0.108	*In vitro* measurement

ADAM, advanced dissolution, absorption and metabolism; B.P, blood-to-plasma partition rate; CL_int_, intrinsic clearance; CL_iv_, *in vivo* clearance; CL_PDM_, clearance passive diffusion maternal blood-placenta cell; CL_PDF_, clearance passive diffusion placenta cell–fetal blood; fa, fraction absorbed; f_u_, fraction of the drug unbound in plasma; HBD, hydrogen bond donor; ka, first-order absorption rate constant; K_p_, tissue-plasma partition coefficient; LogP, log of the octanol-water partition coefficient; MW, molecular weight; P_app_,_Caco-2_, apparent permeability coefficient using Caco-2 cells, P_*eff,*_ effective permeability in man; pKa, acid dissociation constant; PSA, polar surface area; V_ss_, volume of the distribution at steady state.

### 2.2 Model evaluation

For model evaluation, predicted plasma concentration time profiles were qualified against clinical observed data collected in literature for intravenous and oral administration of different doses. Population simulations were performed for 100 (10 trials × 10 individuals) virtual adult individuals, while matching age and weight to the demographics reported for the clinical study in literature (see Supplementary Table S1). The geometric mean fold error (GMFE) was calculated for the ratio of the predicted over observed concentration profile for Area Under Curve (AUC) and maximum concentration (C_max_) (Eq. 1). The model was accepted when the C_max_ and AUC GMFE was within a two-fold error margin (0.5–2.00) (Xu et al., 2021).

$$GMFE=10^{\left(\sum \left|\log_{10}\left(\frac{pred\;PK\;parameter}{obs\;PK\;parameter}\right)\right|\right)/n}$$ (1)

In addition, to verify the predictive performance of the model, visual predictive checks (VPC) were used to compare the predicted concentration time profiles with observed plasma profiles reported for adult reference individuals. As an acceptance criterion, a maximum of 10% of observations outside the 90% prediction interval (5th-95th percentile) was used (European Medicine Agency, 2018).

### 2.3 PBPK model of sildenafil in pregnant women

The PBPK model developed in reference individuals was then used to establish a PBPK model in pregnant women. The Sim-Pregnancy population module in the Simcyp simulator was selected to consider all the physiologic changes (e.g., blood flow, body weight, enzyme expression, and albumin) in the population for the gestational age of 38 weeks. The maternal model was coupled with the fetal model using the permeability-limited placental model. Data from *ex vivo* human placenta experiments were used to inform the transplacental kinetics (Russo et al., 2018). These data were based on a previously developed model describing the *ex vivo* transfer through the placenta and to estimate the cotyledon clearance, CL_cot_ 0.152 L/h (Hoeben et al., 2018).

The transplacental permeability clearance was calculated using the Simcyp simulator toolbox with input of the CL_cot_ = 0.152 L/h/cotyledon, and assuming 17 cotyledons/placenta. Hence, the calculated passive diffusion clearance between mother and fetus (CL_PDM/PDF_), and used as input value in the Simulator, was calculated to be 0.0038168 L/h/mL placenta (Table 1).

The predicted PK profile for pregnant women was compared with the data provided in the clinical trial study RIDSTRESS (“Reducing the Incidence of Fetal Distress with Sildenafil Citrate”) conducted at the Mater Mother’s Hospital in Brisbane Australia. In the study, women of 18–50 years of age at gestational week equal or greater than 37 weeks, in early labor received *via* oral administration 50 mg of sildenafil citrate in fasted state. The maternal blood samples were collected 1–3 h after the dose administration. In addition, cord blood samples were collected right after the delivery (Turner et al., 2020). The samples were collected in Mater Mother’s Hospital in Brisbane Australia and shipped to BioNotus (Niel, Belgium) for analysis, as described below.

The established pregnancy PBPK model was subsequently used for simulating concentration-time profiles in pregnant women, at the following dosing regimens: doses of 50, 75, 100, 130, 150, 175, 200, 250, 300, 320, and 360 mg/day of SIL (immediate release tablets), administered as t.i.d. (three times a day). The simulations included predicting fetal concentration time profiles, allowing determination of the dosing regimen necessary to achieve fetal exposure within the (reference) therapeutic window of 47–500 ng/mL (Barst et al., 2012; Russo et al., 2016). However, it should be noted that the boundaries of this therapeutic window assume a *fu* of 0.036 applicable in reference individuals. As *fu* values are altered during pregnancy and in the fetal circulation, the final simulations were performed assuming either: 1) The *fu* in fetal plasma predicted by the Simcyp software, or the *fu* in fetal plasma determined experimentally *in vitro* as described below. Prediction of fetal *fu* was achieved by first calculating the K_d_ value for sildenafil binding to albumin with the following equation:

$$Kd=\frac{\left[P\right]ref}{\frac{1}{fu}-1}$$ (2)

Kd=1.68 µM

Where [P] *ref* is the reference protein concentration of 45 g/L, and *fu* = 0.036 in the adult reference population.

Assuming the K_d_ is similar between adult and fetal albumin (JORI et al., 2009), the *fu* in fetal plasma was then calculated based on input of this K_d_ value (1.68 µM) along with the reported albumin concentration in the fetus (36 g/L), as follows:

$$fu=\frac{1}{1+\frac{\left[Albumin\right]}{Kd}}$$ (3)

fu=0.044

### 2.4 Bioanalytical assay

The maternal and fetal concentrations of SIL and DMS from RIDSTRESS study (46 samples) were analyzed using Liquid Chromatography-Mass Spectrometry (LC-MS/MS). The LC-MS/MS system used for the analysis was a UPLC Shimadzu Nexera X2 coupled to a LC-MS8050. The column for the analysis was a Kinetex^®^ XB-C18 2.6 µm, 50 × 2.1 mm, 100 Å. The column temperature was set to 30°C to have a constant and stable environment for the flow, the flow rate was set to 0.3 mL/min. The mobile phase was 0.1% formic acid in water (mobile phase A) and acetonitrile: methanol (50:50 v/v) (mobile phase B), isocratic mode. One µL sample was injected and the run time was 6 min. The MS parameters were optimized to reflect the best sensitivity and signal stability. SIL and DMS transitions were 475.00/58.15 and 461.10/85.20, respectively. The measurement range was 7.8–1,000 ng/mL. A 1/x^2^-weighted linear regression model was used to adequately describe the concentration–peak area relationship. The calibration curve standards and quality control standards were in compliance with bioanalytical guidelines (ICH guideline M10) for this run.

### 2.5 Determination of fraction unbound in fetal plasma samples

To determine the SIL free fraction, fetal plasma samples were equilibrium dialyzed for 4 h at 37°C against blank phosphate buffered saline with the HTDialysis device (Gales Ferry, United States of America) using 12 kDa (molecular weight cut off) membrane dialysis strips. Prior to bioanalysis, the collected PBS and fetal plasma samples after dialysis were diluted in 1:1 with blank plasma or blank buffer, respectively, to create a common matrix (50% plasma in PBS). Next, the samples were precipitated using four volumes of methanol, vortexed, centrifugated and the clear supernatant was transferred into vials for LC-MS/MS analysis.

### 2.6 Bioanalysis of samples after equilibrium dialysis

Assay conditions were the same as described above, with the following modifications. The LC-MS/MS column temperature was set to 40°C and the flow rate was set to 0.45 mL/min. 10 µL sample was injected and the run time was 3 min. The measurement range was 1–200 ng/mL, a 1/x-weighted linear regression model was used. The calibration curve standards and quality control standards were in compliance with bioanalytical guidelines (ICH guideline M10) for this run.

The *fu* was calculated by dividing the SIL concentration in buffer (C_free_) by the SIL concentration in the plasma after equilibrium dialysis.

### 2.7 Probability of target attainment (PTA)

To perform the PTA analyses, the percentages of simulated individuals with average (C_avg_), trough (C_tr_) or peak (C_max_) plasma concentrations equal to or above a prespecified target plasma concentration were calculated for different dosing regimens. Because of the sensitivity of the model simulations to the *fu* values, PTA analyses were performed both for predicted and experimentally determined fetal *fu* values.

## 3 Results

### 3.1 PBPK model in non-pregnant population

**FIGURE 1:**
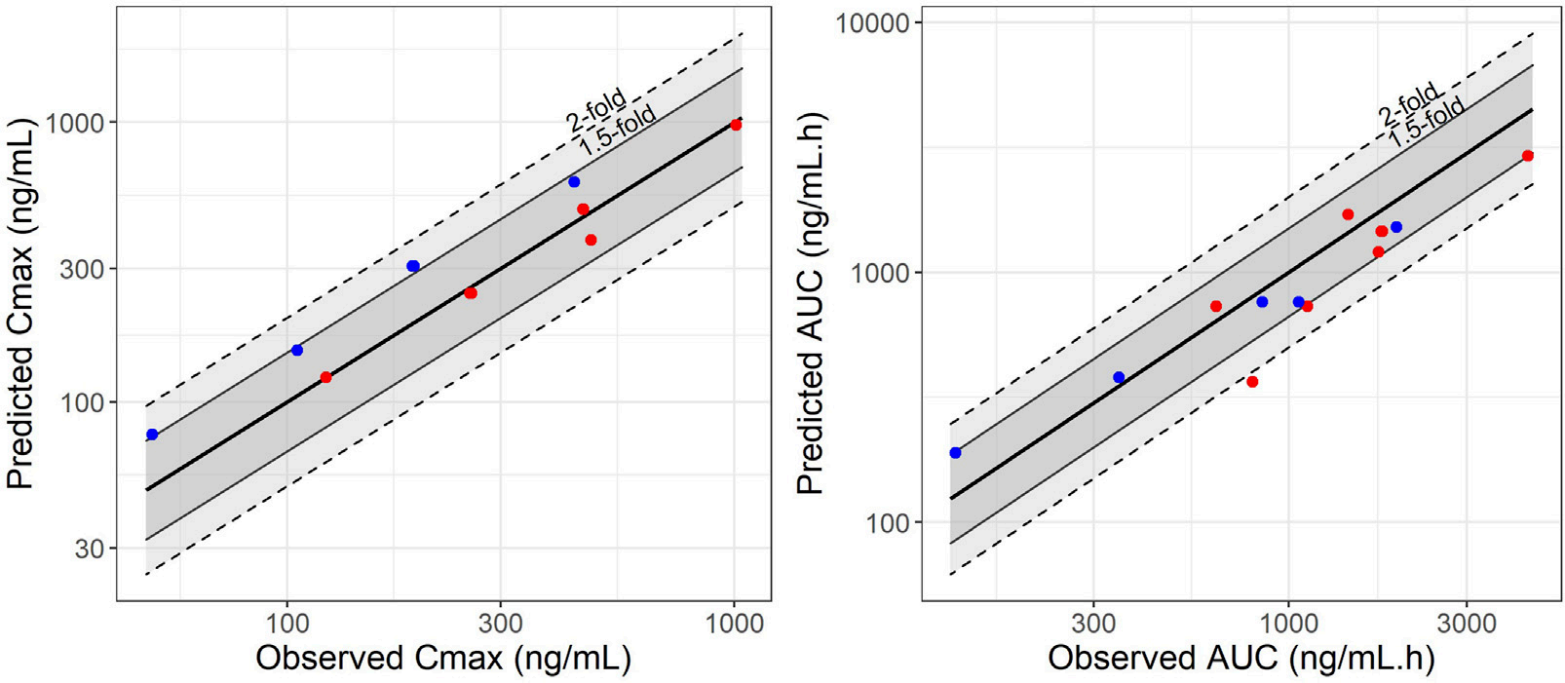
Predictive performance graphs for C_max_ and AUC of sildenafil (represented by red points) and N-Desmethyl-Sildenafil (represented by blue points). A comparison was made between observed and simulated C_max_ and AUC values for adult reference individuals at different dose levels. Solid lines represent the median line of unity, dashed lines and solid lines represent the two-fold and 1.5-fold difference, respectively.

A full-PBPK model was developed for SIL and DMS in adult reference individuals. The GMFE of the predicted over observed ratio for SIL was 0.79 for AUC and 0.99 for C_max_. For DMS GMFE was 0.96 for AUC and 1.5 for C_max_ after intravenous and single oral administration at different doses (25, 50, 100, and 200 mg). The AUC and C_max_ predictions and observed results are summarized in Supplementary Table S1. The predicted GMFE values of SIL were comparable to the observed data within the two-fold range of the observed values. In Figure 1 the predictive performance graph shows that the calculated ratios are mostly between 1.5-fold when compared to the AUC and C_max_ predictions. The VPC showed that the PK profile was well predicted when compared with the observed data collected from literature and are presented in Supplementary Figure S1. The results suggests that the PBPK model developed for adult reference individuals describes well the PK profiles of SIL and DMS and qualifies as a base model to establish a PBPK model in pregnant women.

### 3.2 Fraction unbound in fetus

Equilibrium dialysis was used for the determination of fraction unbound of SIL in selected plasma samples prepared from the cord blood obtained in the RIDTRESS study (Turner et al., 2020). The measured *fu* values are given in Table 2, ranging between 0.067 and 0.167, with a mean (± SD) value 0.108 (±0.041). Out of a total of six samples, one value (0.698) was found to be an outlier and censored.

**TABLE 2:** Summary of unbound fraction of sildenafil in cord blood samples.

	***f*_*u*_**
CB_1_	0.167
CB_2_	0.122
CB_3_	0.073
CB_4_	0.112
CB_5_	0.067
Median	0.112
SD	0.041
Mean	0.108

CB, cord blood; SD, standard deviation.

### 3.3 PBPK in pregnant women and dosing finding

**FIGURE 2:**
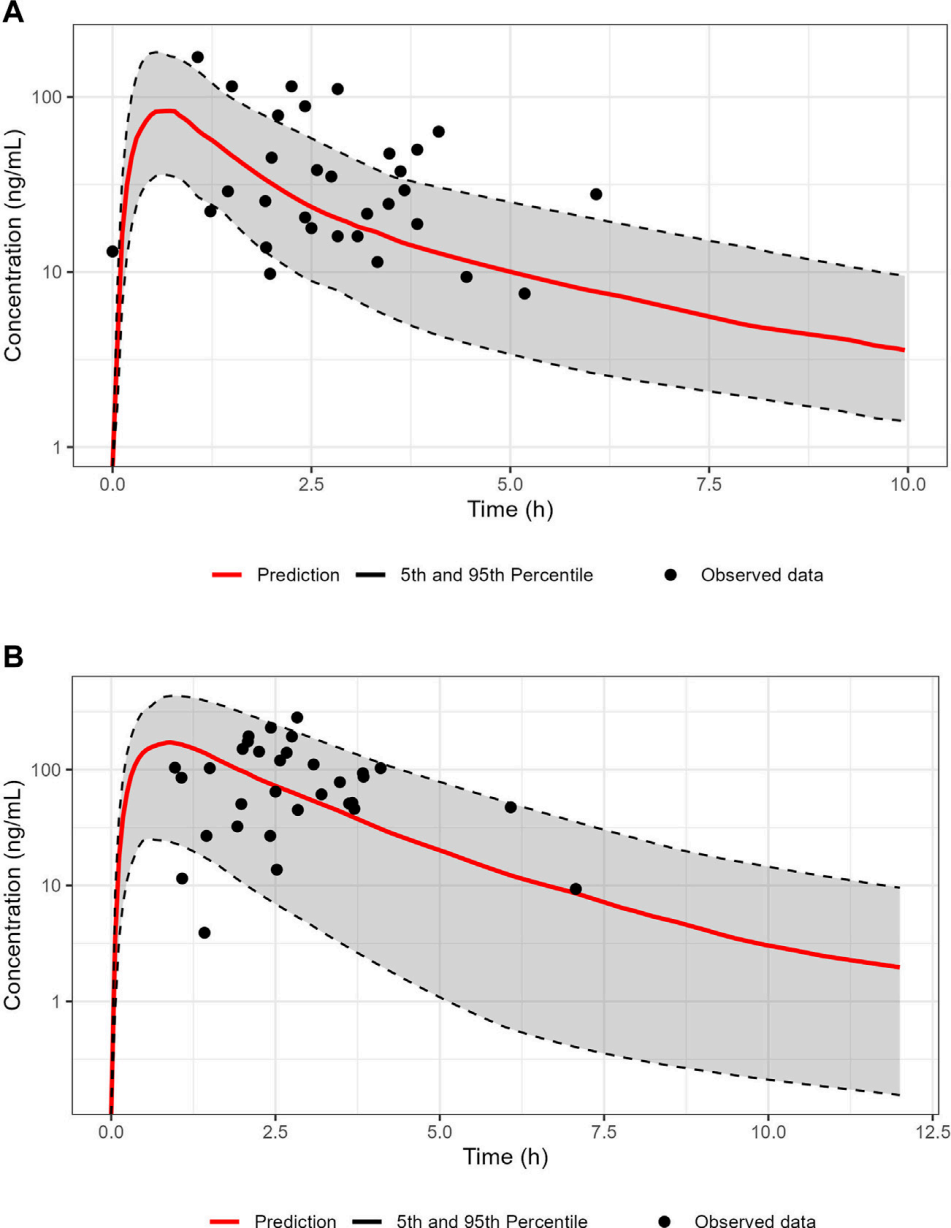
Overlay of the predicted concentration-time profile of sildenafil **(A)** and N-Desmethyl-Sildenafil **(B)** after oral dose of 50 mg under fasted conditions to virtual pregnant women (n = 100; gestational age 38 weeks). The red line represents the median predicted profile while the black dashed lines represent the 5th and 95th percentile profiles. The points represent individual measurements from the RIDSTRESS study.

**FIGURE 3:**
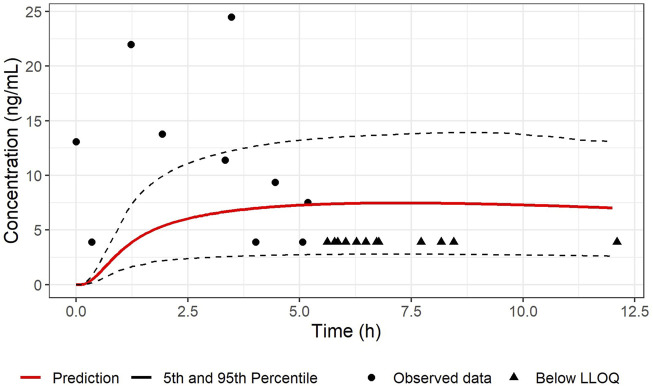
Comparison between the predicted concentration-time profile of SIL in umbilical cord vein plasma and observations after an oral dose of 50 mg sildenafil under fasted conditions in pregnant women (n = 100; gestational age 38 weeks). The red line represents the median predicted profile while the black dashed lines represent the 5th and 95th percentile profiles. The circle shape points represent cord blood measurement from the RIDSTRESS study, and the triangle shape points represents the concentrations below the lower limit of quantitation (LLOQ), represented here at the LLOQ.

The PBPK model as successfully developed and verified for the reference population, was used to establish a PBPK model for pregnant women at 38 weeks in gestation including the fetus. Population simulations for virtual pregnant women were performed and the demographic information was set according to the available clinical data (RIDSTRESS). The predicted plasma concentration-time profile is given in Figures 2, 3 for maternal and umbilical vein plasma concentrations, respectively. The predicted PK profile was compared with the clinical observed data after oral administration of 50 mg in fasted state. The model was evaluated considering the VPCs for both mother and fetus. Given that the RIDSTRESS study was not designed as a PK study (which may have caused additional variability in observed data), the plasma concentration time profiles had a reasonable agreement with the clinical observations. While it was assumed that subjects in the RIDSTRESS trial received a single oral dose of 50 mg sildenafil, the fact that one fetal concentration at t = 0 h was about 13 ng/mL (see Figure 3) strongly suggests that some subjects may have received prior doses. Consistently, seven maternal concentrations were found far above the 90% prediction interval, again indicating that these subjects may have received multiple doses. With only two DMS measurements below the 90% prediction interval, the model provided a good prediction of the concentration time profile of the metabolite.

The pregnancy PBPK model was subsequently used to perform steady-state simulations in populations of 1,000 pregnant women receiving doses of 50, 75, 100, 130, 150, 175, 200, 250, 300, 320, and 360 mg/day, administered as t.i.d. The corresponding predicted maternal and fetal concentrations are shown in Supplementary Figures S2, S3.

**FIGURE 4:**
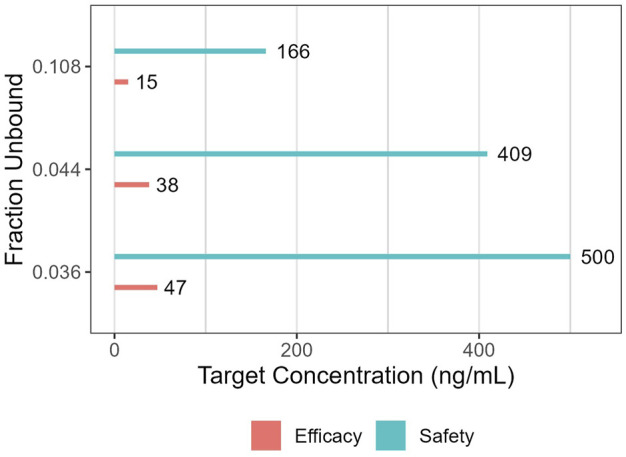
Target concentrations for efficacy and safety when relying on either reference (*fu:* 0.036), experimentally determined (*fu:* 0.108) or predicted (*fu:* 0.044) fraction unbound.

The suitability of maternal and fetal exposures was evaluated using C_avg_ (see Table 3). This was justified given the (very) limited fluctuation of concentrations between troughs and peaks within each steady state dosing interval (irrespective of the percentile). The therapeutic “target” concentration range of 47 ng/mL (efficacy) to 500 ng/mL (toxicity) was used as a reference, but required modification in this special population (because this target range is based on *fu* values of 0.036 reported for reference individuals). Therefore, in a first scenario, this target range was corrected, assuming a predicted *fu* value of 0.044. This resulted in a corrected therapeutic window of 38–409 ng/mL. In a second scenario, the significantly higher (experimentally determined) *fu* value (0.108) was used, yielding binding-corrected values of 15 ng/mL and 166 ng/mL for efficacy and toxicity, respectively (see Figure 4). Furthermore, taking into consideration the interindividual variability in the population, C_trough_ (C_tr_) values at the 5th percentile were also considered for efficacy, while the C_max_ values at the 95th percentile were used for toxicity evaluation (see Table 3).

**TABLE 3:** Simulated steady state average concentrations (C_avg_) at the median (50th percentile), steady state maximum concentrations at the 95th percentile (C_max_) and steady state trough concentrations at the fifth percentile (C_tr_) after administration of 50 mg/day to 360 mg/day, given in three times (t.i.d.), concentrations are given in ng/mL.

Dose(mg/day)	maternal			fetus					
				*f*_*u*_ *in vitro*			*f*_*u*_ predicted		
	C_tr_	C_avg_	C_max_	C_tr_	C_avg_	C_max_	C_tr_	C_avg_	C_max_
**50**	1.04	10.80	76.82	2.23	5.75	11.74	5.21	13.85	28.51
**75**	1.57	16.27	115.70	3.36	8.66	17.78	7.85	20.86	42.94
**100**	2.10	21.69	154.20	4.48	11.55	23.57	10.46	27.81	57.25
**130**	2.72	28.18	200.40	5.81	15.06	30.61	13.59	36.12	74.38
**150**	3.15	32.54	231.40	6.71	17.33	35.35	15.69	41.70	85.89
**175**	2.84	37.94	269.80	7.83	20.21	41.22	18.30	48.64	100.14
**200**	3.25	43.33	308.20	8.94	23.08	47.10	20.09	55.56	114.40
**300**	6.29	65.08	462.80	13.42	34.72	70.70	31.39	83.42	171.77
**320**	6.66	68.99	490.50	14.23	36.74	74.94	33.27	88.43	182.08
**360**	7.55	78.10	555.30	16.11	41.59	84.86	37.67	99.35	206.13

C_tr_, trough concentration; C_avg_, average concentration at steady state; C_max_, maximum concentration; *f*_*u*_, fraction unbound.

When simulations were performed based on the *in vitro* measured *fu* value (0.108), and considering only the median C_avg_ as PK metric, doses starting at 130 mg/day (C_avg_ of 15.06 ng/mL), are considered above the proposed efficacy target and not exceeding (C_max_ of 30.61 ng/mL) the safety target. However, at this dose the C_tr_ at the 5th percentile (5.81 ng/mL), is well below the efficacy target (15 ng/mL). Given the limited fluctuation (C_tr_/C_max_) in SIL concentrations throughout dosing intervals, also the average concentration at the 5th percentile is well below the efficacy target. Supplementary Figure S3 indeed illustrates that about 50% of the population is outside of the therapeutic range when dosing at 130 mg/day. Requiring C_tr_ > efficacy target in at least 95% of the population, doses starting at 360 mg/day (C_avg_ 41.59 ng/mL; C_tr_ 16.11 ng/mL; C_max_ 84.86) are above the efficacy target, and not exceeding the safety target.

The results obtained with simulations using the predicted *fu*, and first considering only C_avg_, doses starting at 150 mg/day (C_avg_ = 41.70 ng/mL), are above the efficacy target (38 ng/mL) and not exceeding the safety target (C_max_ = 85.89 ng/mL). However, at this dose level the 5th percentile of C_tr_ (or C_avg_) does not reach the efficacy target (C_tr_ = 15.69 ng/mL). With the maximum dose simulated of 360 ng/mL, the 5th percentile C_tr_ is (slightly) under the efficacy target (C_tr_ = 37.67 ng/mL < 38 ng/mL), but the 95th percentile C_max_ of 206.13 ng/mL is still under the safety threshold (409 ng/mL). Doses above 360 mg/day were not considered in simulations, since at this dose level the maternal exposure considering C_max_ at the 95th percentile was above the safety threshold (500 ng/mL), Supplementary Figure S2H; Table 3.

PTA analyses were performed to identify dosing regimens achieving the targeted plasma concentrations in at least 95% of the population. The PTA analyses were carried out by comparing simulated steady state C_avg_, C_tr_ and C_max_ values obtained at different dosing regimens with thresholds for efficacy and toxicity, considering both scenarios for obtaining fetal *fu* values (predicted *versus* measured). The results showed a probability of reaching the target concentration in at least 95% of the population at 360 mg/day when *fu in vitro* is considered. However, when *fu* predicted is assumed, the range is only achieved in at least 95% of the population for C_max_ and C_avg_, while 93.5% were above the target for C_tr_ (see Supplementary Figure S4).

## 4 Discussion

SIL is a potent vasodilator used in treatment of erectile dysfunction and pulmonary hypertension in adults (Ghofrani et al., 2006). In recent years, there is a growing interest in the use of SIL during pregnancy to prevent pulmonary hypertension in fetuses with CDH (de Bie et al., 2022). Animal studies have shown that SIL has a potential therapeutic effect in fetuses with congenital diaphragmatic hernia, rescuing peripheral pulmonary vasculature and airway complexity (Luong et al., 2011; Russo et al., 2016; Kashyap et al., 2019; de Bie et al., 2021a). *Ex vivo* human placenta perfusion experiments with SIL confirmed a high rate of transfer across the human placenta (Russo et al., 2018). Taken together, these findings suggest that maternally administered SIL during pregnancy can reach the fetus to exert its putative therapeutic effect. Importantly, these quantitative transplacental clearance data can be exploited to predict fetal exposure to maternally administered SIL with modelling and simulation. Hence the aim of this study was to exploit a PBPK modelling approach for predicting an adequate maternal dosing regimen of SIL resulting in therapeutic fetal exposure. It is important to note that it was the specific intention of the present endeavor to determine a therapeutic dosing strategy exclusively for fetuses with CDH, and explicitly not for other fetal conditions. Of note, results from the STRIDER trial, which was designed to evaluate the use of sildenafil in fetal growth restriction, revealed increased perinatal mortality in one but not all trial centers, causing early trial termination (Groom et al., 2018; Hawkes, 2018; Pels et al., 2020). Nevertheless, as repeatedly commented by (Russo et al., 2019b; 2022) the negative findings in the STIDER trial should not be simply extrapolated to other fetal conditions, including CDH.

Successful verification of the PBPK model for sildenafil and its metabolite in adult reference populations provided an adequate starting point for establishing the pregnancy PBPK model, allowing the subsequent prediction of maternal and fetal SIL exposure during pregnancy. With a PBPK modelling approach, pronounced changes in physiology as well PK of the pregnant population can be accounted for (Abduljalil et al., 2022; Silva et al., 2022). For instance, a remarkable change during pregnancy is the 60% increase in CYP3A4 activity (Hebert et al., 2008). This was reflected in the significantly higher SIL apparent clearance (CL/F) during pregnancy as compared to reference individuals. Importantly, to adequately inform the transplacental passage of SIL in the PBPK model, the previously generated *ex vivo* human placenta data were leveraged.

Comparison of the model-predicted concentration time profiles in pregnant women with observations obtained in the RIDSTRESS study (Turner et al., 2020) revealed an acceptable predictive performance for SIL and DMS in pregnant women. As mentioned in the results section, seven maternal plasma concentrations positioned relatively far above the prediction interval, whereas only two DMS concentrations were outside (below) the prediction interval. At the fetal side, only a limited number of cord blood samples were above the quantification limit, but predictions were in agreement with some observations above the LLOQ. Fetal DMS concentrations were found to be very low (<LLLQ), which supports the assumptions that DMS does not cross the placenta (based on a more hydrophilic character compared to SIL), and is not generated by the fetus in clinically relevant concentrations. The latter appears consistent with the much lower conversion by (fetal) CYP3A7 compared to CYP3A4 (Zane et al., 2015). Based on these comparisons, the pregnancy PBPK model for sildenafil was considered adequate to be used for simulation-based dose predictions. Unfortunately, no other PK data from maternal or cord blood samples were available to further support model evaluation. This includes the absence of observations before term gestational age.

Prior to engaging in model-based simulations, one specific aspect of the pregnancy PBPK model that deserves particular attention is the dual impact of altered fetal (and maternal) protein binding as compared to the reference population. First, such changes in *fu* are likely to affect the maternal/fetal distribution of SIL. Indeed, a significantly higher free fraction of SIL in the fetal circulation will eventually result in lower total concentrations for the same unbound concentration at equilibrium. Secondly, a significant increase in *fu* in the fetus requires a reevaluation of SIL efficacy and safety levels, expressed as total concentrations.

To obtain accurate values for the altered SIL *fu* values, two approaches were applied. In one approach, the fraction unbound was calculated based on the change in albumin concentration, thereby assuming no changes in affinity for SIL (i.e., K_d_ remains unchanged). This is likely a valid approach to calculate the maternal *fu* during pregnancy (because of decreasing albumin levels during pregnancy). However, the reliability of this approach for predicting the fetal SIL *fu* value may be questioned. For this reason, and in the absence of a fetus-specific K_d_ value, it was decided to also experimentally determine the fetal *fu* value.

In a first scenario, the PBPK model was built using *fu* values determined by *in vitro* experiments. The simulated fetal concentration time profiles were evaluated according to the (*fu*-adapted) efficacy target of 15 ng/mL (considering the fifth percentile C_tr_) as well as the toxicity threshold of 166 ng/mL (considering the 95th percentile C_max_). In the second scenario, the PBPK model was built using predicted *fu* values. In this case, simulated fetal concentration time profiles were evaluated according to the efficacy target of 38 ng/mL as well as the toxicity threshold of 409 ng/mL. To achieve these targets, required maternal dosing regimens are 300 mg/day when considering fifth percentile C_avg_ values, while even up to 360 mg/day would be needed when considering C_tr_ at the fifth percentile. However, albeit borderline, the 360 mg/day yields maternal peak plasma concentrations above the toxicity threshold. Simulated fetal plasma concentrations never exceeded the toxicity thresholds. Hence a more pragmatic and especially safer approach is to select a dosing regimen based on the median C_avg_ concentration reaching the efficacy target, suggesting 130–150 mg/day administered as t.i.d. as a safe initial dosing regimen. Interestingly, both simulation strategies (based on two different *fu* values) led to similar dose predictions. This similarity was not unexpected, as lower fetal binding leads to less partitioning of sildenafil across the placenta, but also leads to reduced sildenafil target levels when expressed as total concentrations. Therefore, these opposing effects of protein binding were expected to cancel out at least partially.

Remarkably, the *fu* value obtained from *in vitro* experiments was found to be 2.5-fold higher compared to the predicted value. It is worth mentioning here the limitations associated with *fu* values measured *in vitro* in plasma obtained from cord blood samples. The sample size was low (n = 6; one outlier), and intersubject variability was (very) high, suggesting that protein degradation in some samples may not be excluded (potentially explaining the very high *fu* value in the outlier measurement). Although the impact of reduced protein binding turned out to be relatively limited, additional measurements of maternal and fetal fu are warranted to inform more accurately future PBPK models.

In guidance to support the clinicians regarding the initial recommended dosing regimen of SIL, it seems justifiable to take the lowest dosing regimen from the two (*fu*-related) scenario’s presented above. Considering the C_avg_ metric, our findings support t.i.d. Dosing regimens of 130 mg/day and 150 mg/day to be within the therapeutic range. In both cases, the C_tr_ at the fifth percentile is still under the proposed target. However, for safety reasons, an initial dose regimen of 130 mg/day administered as t.i.d., combined with subsequent dose titration by therapeutic drug monitoring (TDM) is recommended (Kang and Lee, 2009). Importantly, actual treatments for CDH will occur in earlier stages of pregnancy as opposed to the setting of the data obtained in the RIDSTRESS clinical study, which was used to verify the pregnancy PBPK model developed here. Thus, considering a progressive increase in sildenafil clearance during the trimesters of pregnancy, the proposed ‘lower’ dosing regimen of 130 mg/day becomes more relevant and justifiable.

In support of this dosing regimen proposed based on PBPK simulations in the present study, it is noteworthy that Samangaya et al. (2009) already reported administration of 80 mg SIL three times a day in pregnant women with preeclampsia. They had followed a titration scheme with initial doses of 20 mg escalating to 80 mg t.i.d. and achieving peak drug concentrations of 271 ng/mL, without any adverse effects reported. (Samangaya et al., 2009), providing further confidence in the proposed regimen of 130 mg/day administered as t.i.d. at least from a maternal safety perspective.

From a (fetal) efficacy perspective, it should be recognized that the reference target concentration (47 ng/mL) for efficacy employed in the present study is based on data regarding efficacy of sildenafil in various pediatric (and adult) populations. This implies that the optimal dose level may change as more relevant data on efficacious concentrations in this specific fetal population become available. Interestingly, the two sheep studies in this field have both demonstrated effects of sildenafil at fetal concentrations well below the currently used therapeutic range. Both an acute pulmonary vasodilatory effect (de Bie et al., 2021b) and an effect on neonatal cardiopulmonary hemodynamics (suggesting a longer-term effect on pulmonary vascular development) (Kashyap et al., 2019) were observed at concentrations that are currently assumed to be ‘subtherapeutic’. It is therefore possible that the required fetal sildenafil concentration to achieve the desired developmental effect is lower than the concentration required to achieve acute vasodilation in neonates. Consistently, based on the data published (Ballard et al., 1998; Kashyap et al., 2019; de Bie et al., 2021a; de Bie et al., 2021b) it is not unlikely that concentrations below those required for achieving 53% PDE5 inhibition are still sufficient to obtain long-term therapeutic effect (due to vascular remodeling) in the fetal population for this specific indication.

In conclusion, this work illustrates the utility of PBPK modelling and simulation as powerful tool for dose finding in special populations, while avoiding unnecessary exposure of vulnerable patients. In the present study, the PBPK modelling approach supported dose finding in pregnant women to achieve therapeutic exposure in the fetus. At the same time, pregnancy-associated physiologic changes were accounted for in the model. Based on our findings, and with specific attention to safety, a dosing regimen of 130 mg/day (administered as t.i.d.) appears recommended as initial prescription of SIL, specifically (and exclusively) in antenatal treatment of congenital diaphragmatic hernia associated with pulmonary hypoplasia and hypertension.

Therapeutic drug monitoring will be required to adapt the initial dosing regimen in specific patients. In addition, these concentration measurements will inform and optimize future PBPK models for this indication and population.

## 5 Contribution to the field

Maternal administration of sildenafil during pregnancy is being evaluated for antenatal treatment of congenital diaphragmatic hernia (CDH) associated with pulmonary hypoplasia and hypertension. However, determination of a safe and effective maternal dosing regimen to achieve adequate fetal exposure to sildenafil remains challenging. In the present study, PBPK modelling and simulation approaches are exploited to determine for the first time a dosing regimen recommendation for treatment initiation with sildenafil, specifically in the CDH indication. Upon treatment initiation, maternal sildenafil dosing should be adapted based on therapeutic drug monitoring.

## Data Availability

The original contributions presented in the study are included in the article/Supplementary Material, further inquiries can be directed to the corresponding author.
