# Supplementary material for: PBPK-based dose finding for sildenafil in pregnant women for antenatal treatment of congenital diaphragmatic hernia
Source: Front Pharmacol. 2023 Mar 14;14:1068153. doi: 10.3389/fphar.2023.1068153 (PMC10043195; doi:10.3389/fphar.2023.1068153)
Supplement: Supplementary file 1 [file Presentation1.pdf]

Supplementary Material

1 Supplementary Figures and Tables

1.1 Supplementary Figures

Supplementary Figure S1a. Overlay of the predicted concentration-time profile of sildenafil (left) and N-Desmethyl-Sildenafil (DMS) (right) after single intravenous administration of 50 mg (a); oral doses of (b) 25 mg (c) 50 mg (d) 100 mg and (e) 200 mg under fasted condition to virtual healthy adult volunteers (n=100). The red line represents the median predicted profile while the black dashed lines represent the 5th and 95th percentile profiles. The points represent the mean observed data (Nichols et al., 2002).

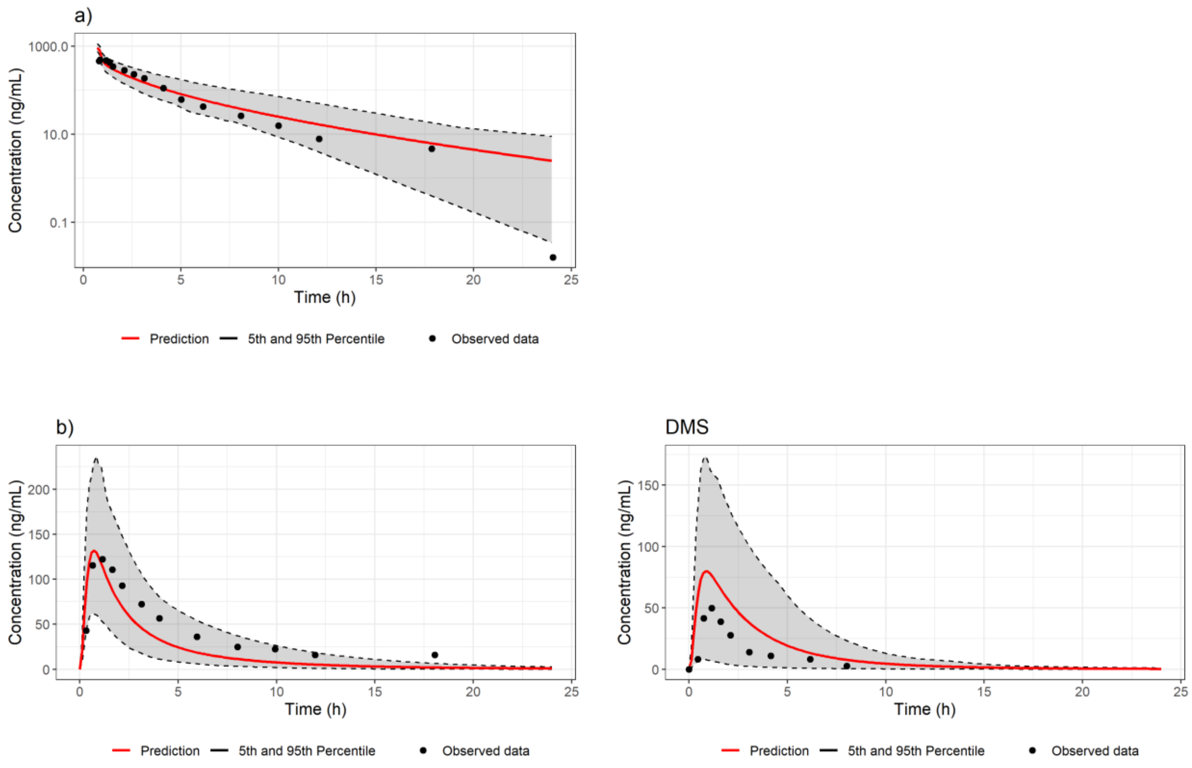

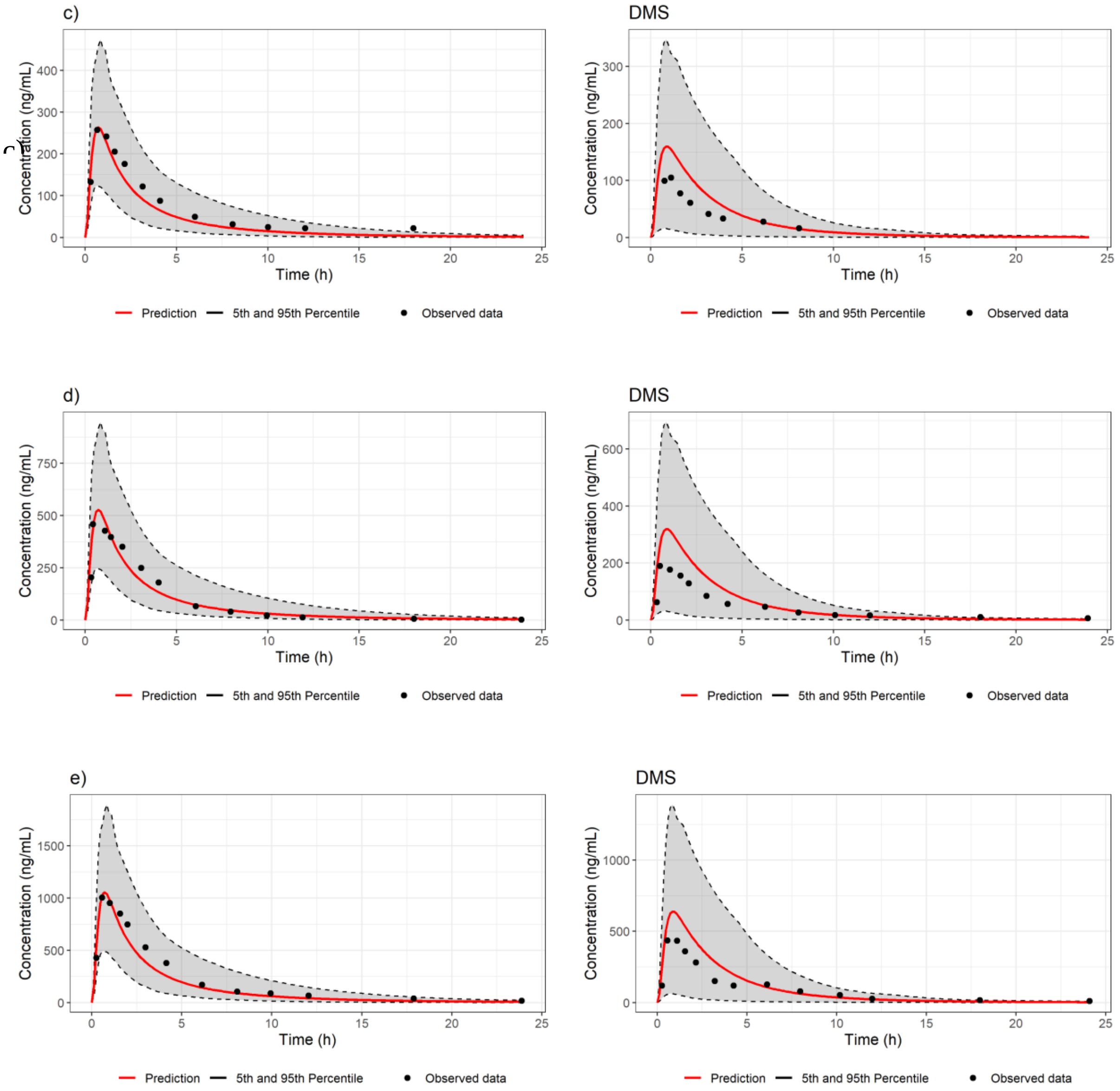
c)
DMS
d)
DMS
e)
DMS
Concentration (ng/mL)
Time (h)
Prediction
5th and 95th Percentile
Observed data

Supplementary Figure S1b. Overlay of the predicted concentration-time profile of sildenafil after multiple dose administration of 80 mg a day under fasted condition to virtual healthy adult volunteers (n=100). The red line represents the median predicted profile while the black dashed lines represent the 5th and 95th percentile profiles. The points represent the mean observed data (Burgess et al., 2008).

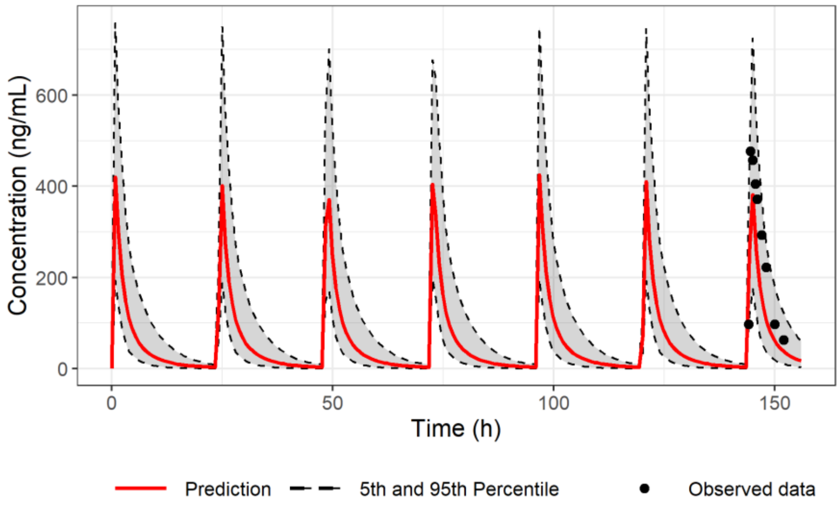

Supplementary Figure S2. Simulated maternal concentration time profiles of sildenafil after oral dose of 50 mg/day (a), 100 mg/day (b), 130 mg/day (c), 150 mg/day (d), 175 mg/day (e), 200 mg/day (f), 300 mg/day (g), 320 mg/day (h) and 360 mg/day (i), administered as t.i.d. in pregnant women. The red line represents the median predicted profile while the dashed lines represent the 5th and 95th percentile profiles. The blue lines represent the (maternal) safety threshold of 500 ng/mL.

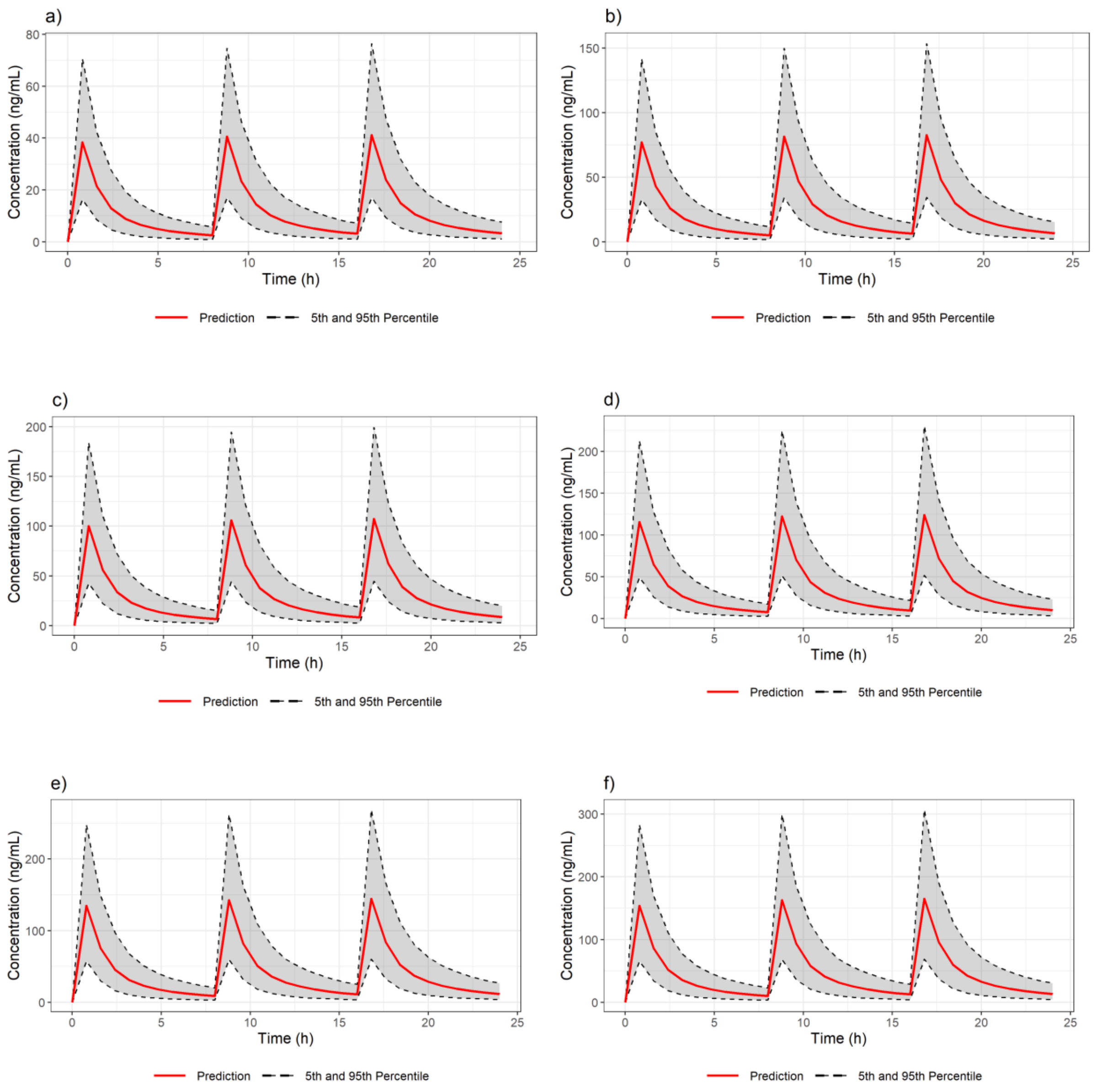

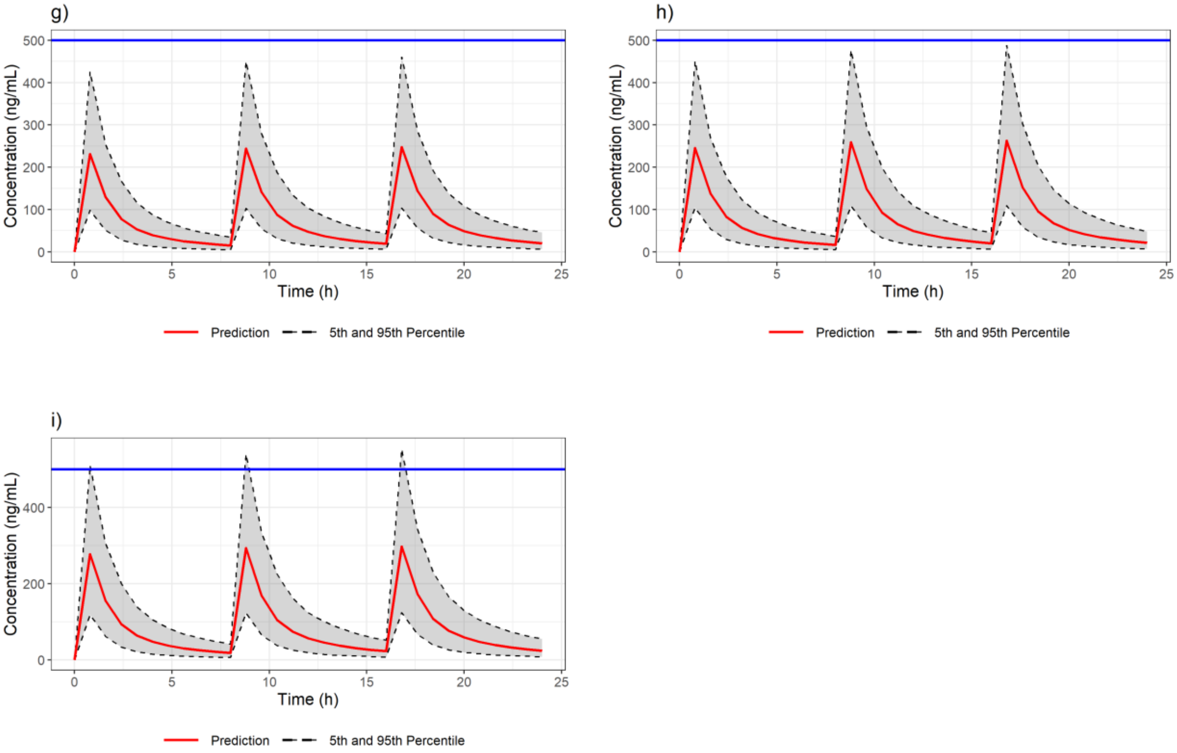
g)
h)
i)
Concentration (ng/mL)
Time (h)
Prediction
5th and 95th Percentile

Supplementary Figure S3. Simulated fetal concentration time profiles of sildenafil after oral dose of 50 mg/day (a), 100 mg/day (b), 130 mg/day (c), 150 mg/day (d), 175 mg/day (e), 200 mg/day (f), 300 mg/day (g), 320 mg/day (h) and 360 mg/day (i), administered as t.i.d. in pregnant women. The red line represents the median predicted profile while the dashed lines represent the 5th and 95th percentile profiles. The blue lines represent the efficacy targets of 15 ng/mL assuming the fraction unbound (f_u) measured *in vitro*, or of 38 ng/mL assuming the *in silico* predicted fraction unbound.

f_u *in vitro* | f_u predicted

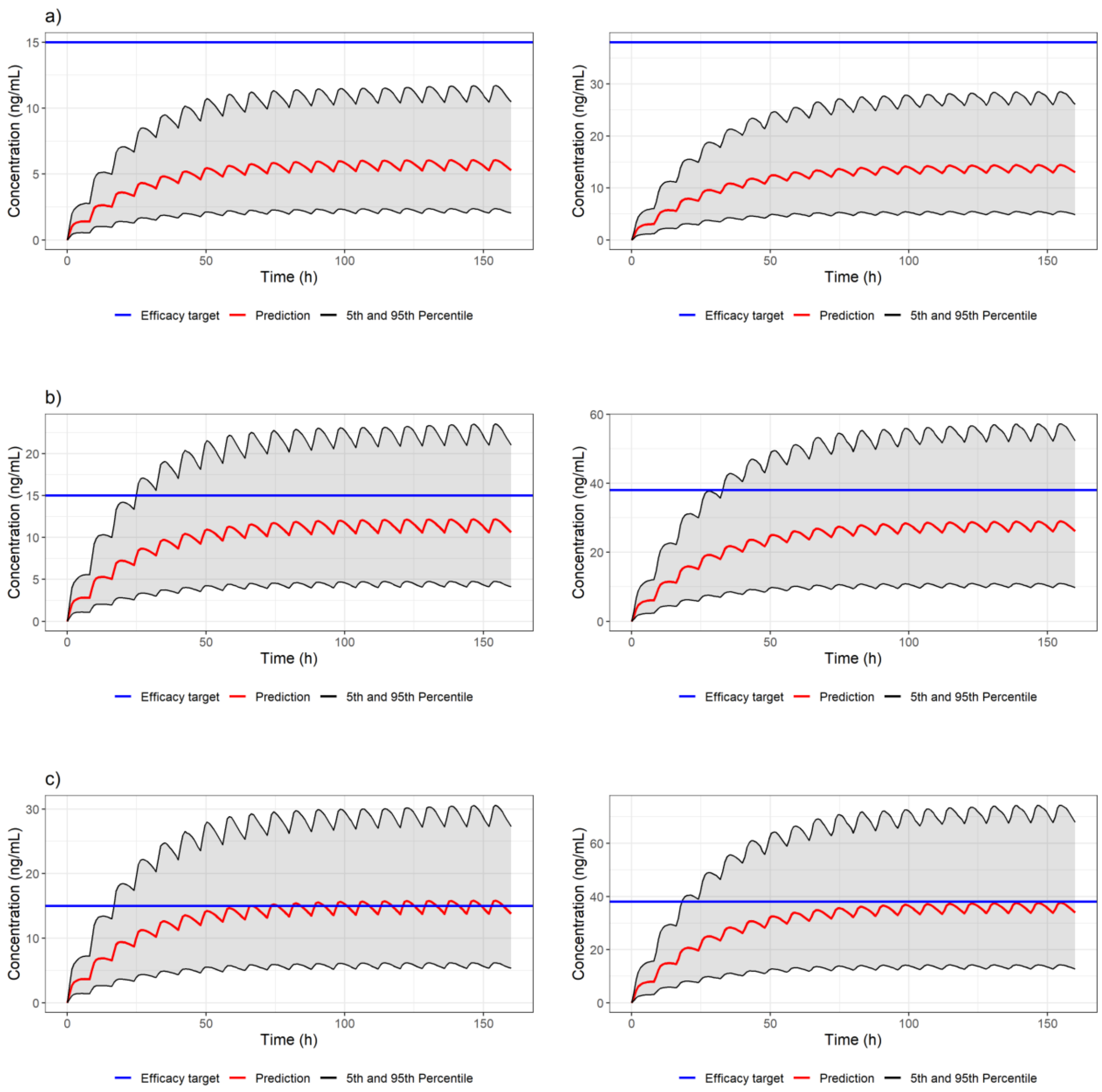

d)

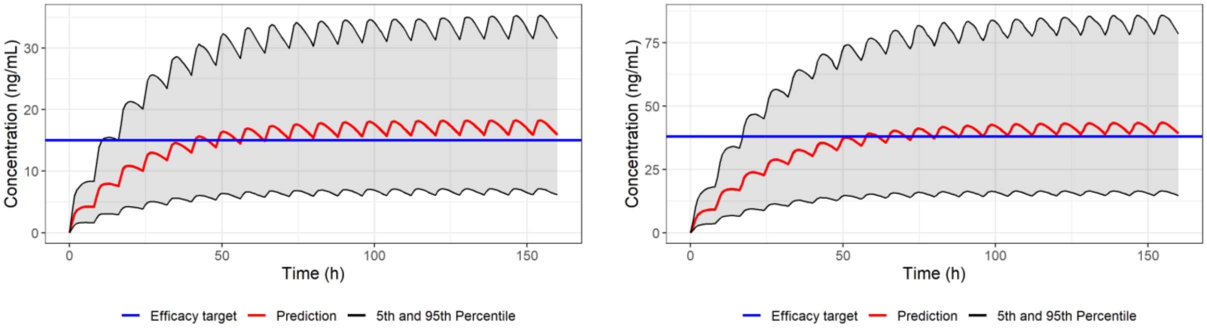
Concentration (ng/mL)
Time (h)
Efficacy target
Prediction
5th and 95th Percentile

e)

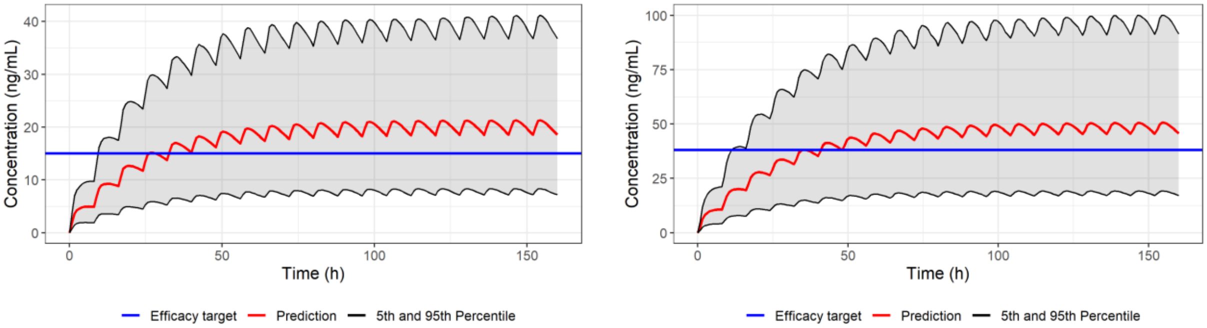
Concentration (ng/mL)
Time (h)
Efficacy target
Prediction
5th and 95th Percentile

f)

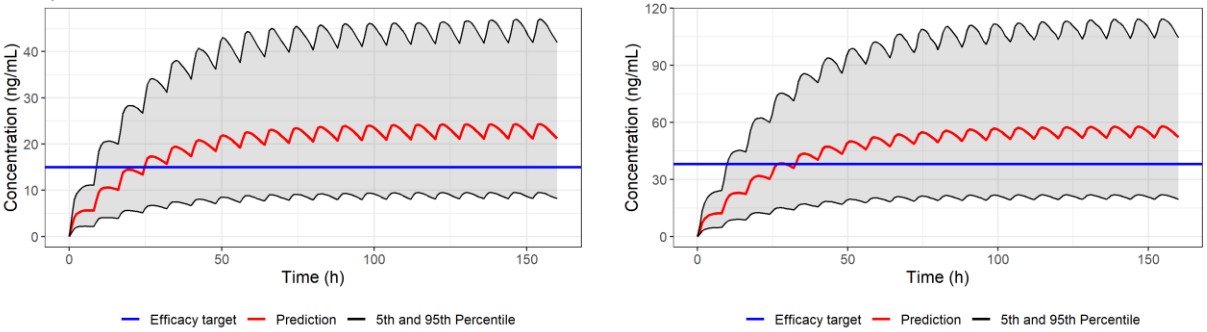
Concentration (ng/mL)
Time (h)
Efficacy target
Prediction
5th and 95th Percentile

g)

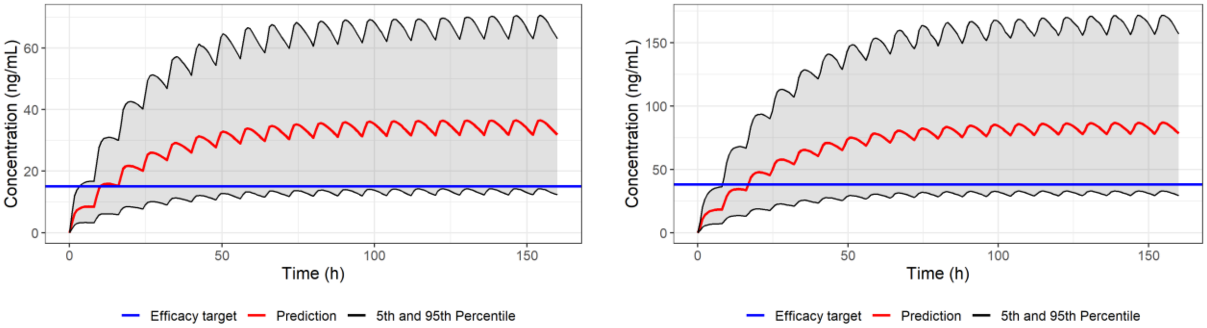
Concentration (ng/mL)
Time (h)
Efficacy target
Prediction
5th and 95th Percentile

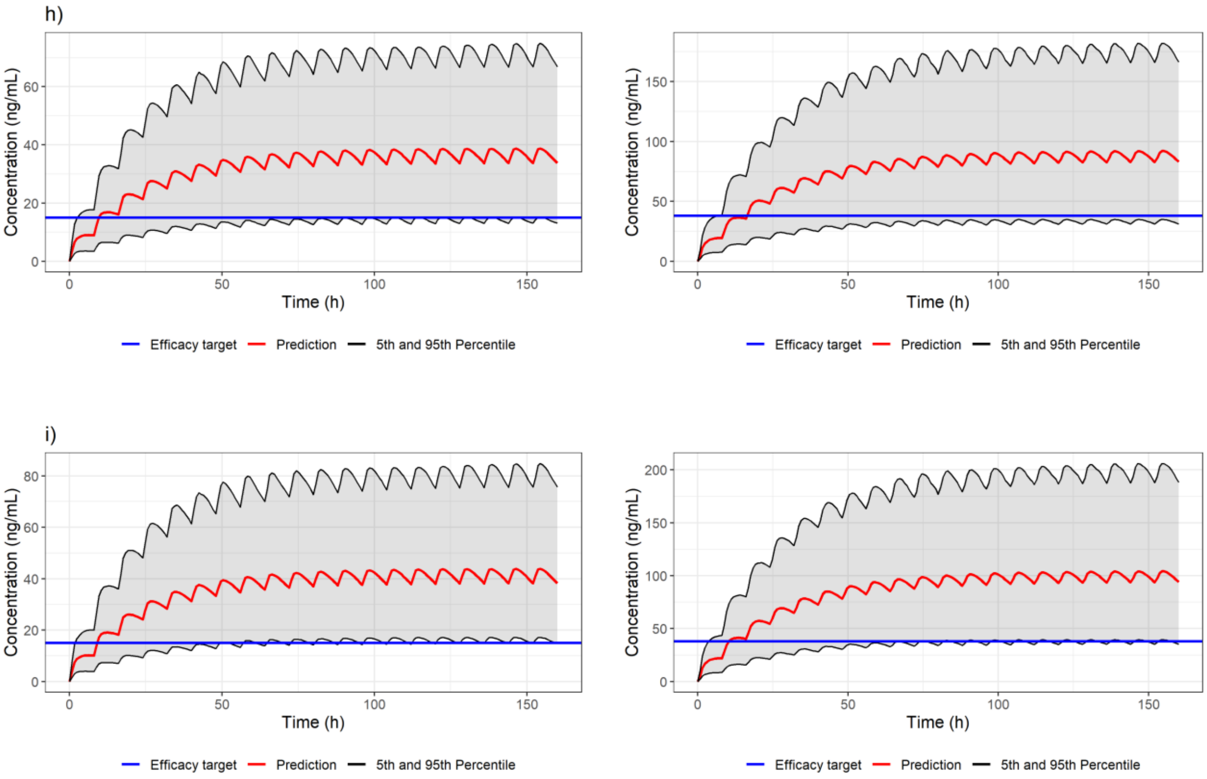

Supplementary Figure S4. Probability of target attainment curves for different sildenafil dosing regimens (50-360 mg/day), considering fetal C_{avg} (c, d), C_{trough} (e,f) or C_{max} (a,b) concentrations, and based on either a measured (0.108; left column) or predicted (0.044; right column) fraction unbound in the fetus.

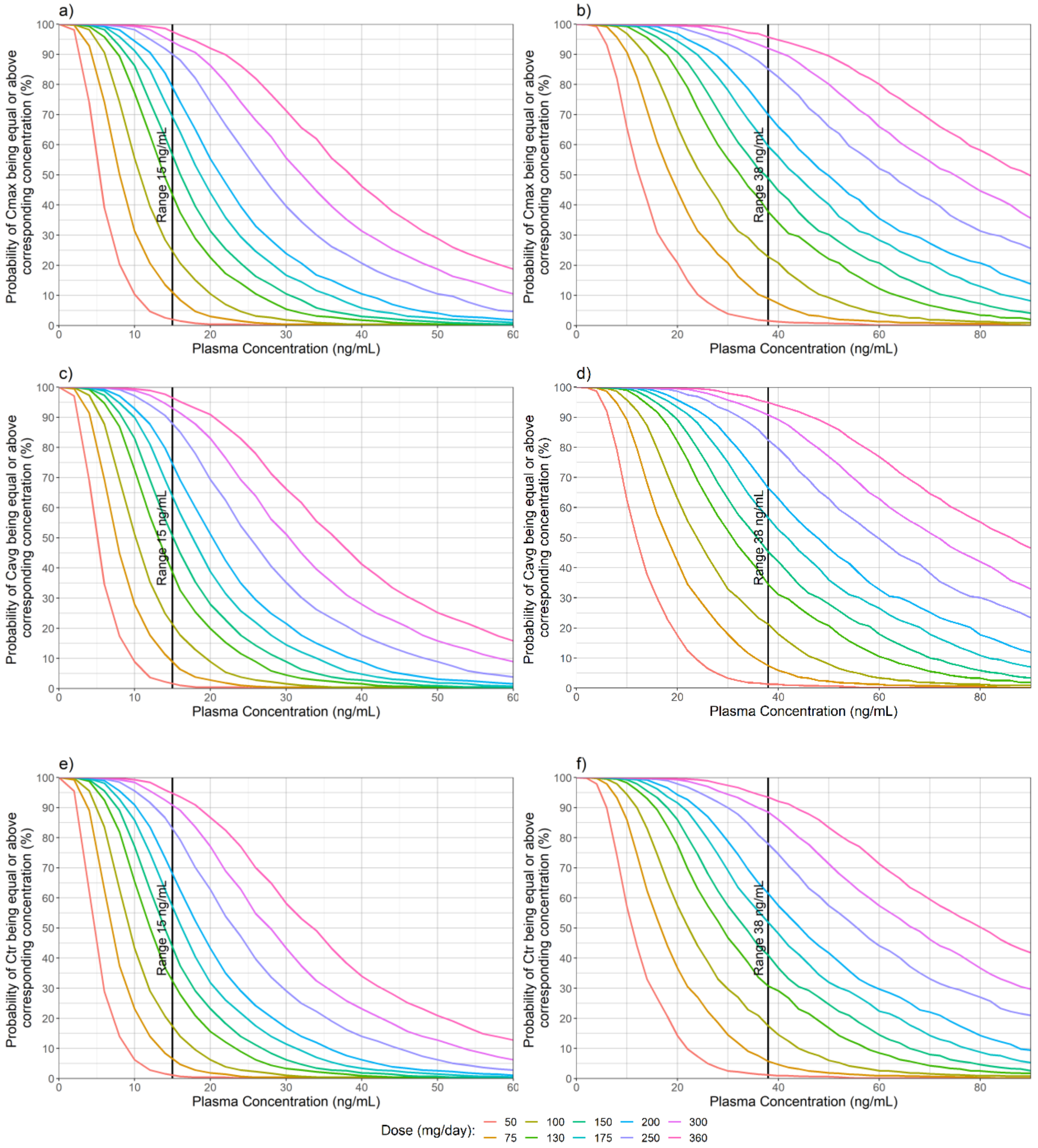

1.2 Supplementary Tables

Supplementary Table S1- Ratio between the predicted and observed pharmacokinetic parameters of sildenafil and N-Desmethyl-Sildenafil in different doses and regimen in adult reference individuals as reference population.

Study ID	Dose (mg)	Route	AUC$_{obs}$	AUC$_{pred}$	Ratio	Cmax$_{obs}$	Cmax$_{pred}$	Ratio	Reference
**Sildenafil**									
Nichols 2002	50	IV	1443.05	1705.40	1.18	-	-	-	(Nichols et al., 2002)
Nichols 2002	25	PO	797.68	366.11	0.46	122.17	122.44	1.00	(Nichols et al., 2002)
Nichols 2002	50	PO	637.24	732.23	1.15	255.92	244.89	0.97	(Nichols et al., 2002)
Nichols 2002	100	PO	1780.26	1464.46	0.82	458.81	489.78	1.07	(Nichols et al., 2002)
Nichols 2002	200	PO	4374.66	2928.93	0.67	1006.79	979.55	0.97	(Nichols et al., 2002)
Gupta 2005	100	PO	1767.90	1464.46	0.83	458.16	489.78	1.07	(Gupta et al., 2005)
Burgess 2006	80 (t.i.d.)	PO	1739.50	1210.98	0.70	477.43	379.20	0.79	(Burgess et al., 2008)
**N-Desmethyl-Sildenafil**									
Nichols 2002	25	PO	127.55	190.21	1.49	49.86	76.48	1.53	(Nichols et al., 2002)
Nichols 2002	50	PO	349.21	380.43	1.09	105.26	152.97	1.45	(Nichols et al., 2002)
Nichols 2002	100	PO	1063.00	760.85	0.72	190.21	306.00	1.61	(Nichols et al., 2002)
Nichols 2002	200	PO	1940.91	1521.71	0.78	437.67	611.90	1.40	(Nichols et al., 2002)
Gupta 2005	100	PO	847.39	760.39	0.90	191.98	305.95	1.59	(Gupta et al., 2005)

AUC: area under curve; Cmax: maximum concentration; IV: intravenous administration; Obs: observed; PO: oral administration; Pred: predicted.
